# Characterization and comparative evaluation of wound healing potential of Ajugarin I and *Ajuga bracteosa* Wall. ex Benth

**DOI:** 10.3389/fchem.2023.1325578

**Published:** 2024-01-31

**Authors:** Yusra Wasti, Durdana Muntaqua, Muhammad Majid, Iffat Naz, Aroosa Zafar, Safir Ullah Khan, Syeda Tayyaba Batool Kazmi, Tofeeq Ur Rehman, Nadeem Irshad, Humaira Fatima

**Affiliations:** ^1^ Department of Pharmacy, Faculty of Biological Sciences, Quaid-i-Azam University, Islamabad, Pakistan; ^2^ Shifa College of Pharmaceutical Sciences, Shifa Tameer-e-Millat University, Islamabad, Pakistan; ^3^ Faculty of Pharmacy, Hamdard University, Islamabad, Pakistan; ^4^ Department of Biology, Science Unit, Deanship of Educational Services, Qassim University, Buraidah, Saudi Arabia; ^5^ Hefei National Laboratory for Physical Sciences at the Microscale, School of Life Sciences, University of Science and Technology of China, Hefei, China

**Keywords:** Ajuga species, Ajugarin I, polyphenols, high pressure liquid chromatography, wound healing

## Abstract

*Ajuga bracteosa* (family: Lamiaceae), commonly known as kauri booti, is an important ethnomedicinal plant. The current research was conducted to appraise and compare the *in vitro* antioxidant and antibacterial profiles as well as *in vivo* wound healing potentials of Ajugarin I and *A. bracteosa* extract. Ajugarin I and polyphenols in *A. bracteosa* were enumerated by reversed-phase high-performance liquid chromatography analysis that confirmed significant amounts of Ajugarin I (2.2 ± 0.02 μg/mg DW) and other phenolic compounds (14 out of 17 standards). *A. bracteosa* (374.4 ± 0.20 µg AAE/mg of DW, 201.9 ± 0.20 µg AAE/mg of DW, 87 ± 0.30%) showed a higher antioxidant profile compared to Ajugarin I (221.8 ± 0.50 µg AAE/mg of DW, 51.8 ± 0.40 µg AAE/mg of DW, 27.65 ± 0.80%) with 1.86-, 3.89-, and 3.15-fold greater activity in ferric reducing antioxidant power, total antioxidant capacity, and free radical scavenging assays, respectively. Likewise, *A. bracteosa* showed antibacterial activity against 3/5 strains (MIC 25–200 μg/ml) than Ajugarin I (2/5 strains; MIC 50–200 μg/ml). Hemolytic (<2% hemolysis) and dermal toxicity tests rendered both samples non-toxic. Additionally, *A. bracteosa* (100 ± 2.34% at day 12; 9.33 ± 0.47 days) demonstrated 1.11- and 1.24-fold higher percent wound contraction and epithelization time, respectively, than Ajugarin I (95.6 ± 1.52% at day 12; 11.6 ± 0.47 days) as assessed by an excision wound model in mice. Histopathological examination further reinforced the better wound healing potential of *A. bracteosa* with good epithelization, collagen synthesis, fibroblast proliferation, and revascularization. Briefly, we endorse the significant comparative antioxidant, antibacterial, and wound healing activities of *A. bracteosa* and Ajugarin I and present these as prospective candidates for wound healing drugs.

## 1 Introduction

Skin wounds represent affronts to the body’s anatomical barrier, eliciting physiological modifications to counteract the imposed injury. Wounds damage skin integrity due to external stimuli; for example, surgery, burns, cuts, bedsores, etc. Wounds are generally categorized as open or closed based on exposure to the external environment. Open wounds are characterized by damage to the mucous membrane of skin, which allows the particles to move inside the tissue (Zomer and Trentin, 2018). Open wounds are usually infected since the skin is exposed to external stimuli like dirt, bacteria, and contaminants. On the other hand, closed wounds are not exposed to the external environment, have intact skin layer with underlying injury and restore without infection (Bowler et al., 2001). Since skin regenerates frequently and is flexible with a good supply of blood, wounds therefore have the capacity to heal based on the extent of the injury (Nichols and Katiyar, 2010).

Wound healing involves different phases, i.e., homeostasis, inflammation, proliferation, and remodeling. Homeostasis is the first step of wound healing, which plugs the injury with clot formation. The next step is inflammation, during which the inflammatory mediators and macrophages move toward the site of the wound (Rodrigues et al., 2019). Macrophages help in the removal of dead cells and debris. Neutrophils accumulate in wound areas and induce free radical generation, clear microbes, and produce inflammatory cytokines and growth factors that progress the inflammatory phase (Ebaid, 2014). Subsequently, the inflammatory phase is shifted into the proliferative phase that initiates the formation of new blood vessels and fibroblasts. The resident fibroblasts differentiate into myofibroblasts creating the margins of wounds and the depositing of extracellular matrix. The composite of fibroblasts, vascular endothelial cells, connective tissue, macrophages, collagen, and fibrin constitute the granulation tissue that covers the skin by secondary intention. Granulation tissue appears light red or dark pink in color due to enhanced blood flow in newly formed blood vessels. Simultaneously, re-epithelization occurs which involves the proliferation of epidermal stem cells from the basement membrane and stem cells for skin appendages like sweat glands, sebaceous glands, and hair follicles. Lastly, the skin is remodeled, restoring barrier function and near-normal tensile strength along with the formation of a fibrotic scar that covers the wound (Yamaguchi and Yoshikawa, 2001; Rodrigues et al., 2019). Wound care protects wounds from infections and supports the healing process. Multiple complementary and alternative medicines have been used traditionally to facilitate wound healing (SHAIKH et al., 2019). For example, in traditional Persian medicine, the genus *Ajuga* has been used to treat jaundice, gout, injuries, and sciatica (Rahiminiya et al., 2021). Among various *Ajuga* species, *Ajuga bracteosa* Wall. ex Benth. is used to treat various ailments. *A. bracteosa* is an evergreen herb that belongs to the Labiatae/Lamiaceae family (Kaithwas et al., 2012). It is known as ‘bungle’, ‘jan-i-adam’, and ‘kauri booti (bitter taste)’ in English, Kashmiri, and Punjabi/Hindi, respectively (Ganaie et al., 2017). Traditionally, the leaves are used for headaches, diabetes, and fever and as a blood purifier; the bark is utilized to treat acne and the whole plant is prescribed for abdominal pain (Ahmed and Chaudhary, 2009), swollen wounds, insect bites, and as an astringent (Hussain et al., 2016). Multiple phytochemicals are isolated from *A. bracteosa* that include phytoecdysteroids, flavonoids, neo-clerodane diterpenoids, and glycosides (Luan et al., 2019). These plant compounds have been reported as having anticancer (Chander, 2018), anti-inflammatory (Zhang and Tsao, 2016), antiarthritic (Kaithwas et al., 2012), antibacterial, and antiviral activities (Orhan et al., 2012). A recent study has reported the wound-healing capacity of *A. bracteosa* nanoparticles (Andleeb et al., 2022). However, it is not clear whether this activity is solely attributed to Ajugarin I or not. Hence, in the present research, we intended to confirm the traditional use of *A. bracteosa* in wound care and compare the wound healing potential of crude *A. bracteosa* with the Ajugarin I, a major phytoconstituents in *Ajuga*. Our study provides a significant analysis of wound healing activity of *A. bracteosa* and is the first report on the healing benefits of Ajugarin I in the animal model.

## 2 Materials and methods

### 2.1 Chemicals and reagents

Ajugarin I was acquired from Prof. Ihsan-ul-Haq, Quaid-i-Azam University (QAU), Islamabad, Pakistan (Zahra et al., 2017). Solvents, chemicals, and salts were procured from Sigma Aldrich (United States) unless stated otherwise.

### 2.2 Animals

Balb/c mice (25–30 g) with an age of 6–7 weeks were used for *in vivo* wound healing studies. Mice were obtained from the National Institute of Health (NIH), Islamabad, Pakistan, and were retained in the animal facility of the Department of Pharmacy, QAU, Islamabad. All animals were provided water and food *ad libitum* and were housed in standardized environmental conditions, i.e., 12/12 light/dark cycles with temperature and humidity maintained at 22°C and 60%, respectively.

### 2.3 Ethical statement

The Bioethical Committee of QAU, Islamabad, approved all investigations involving animals and human blood in the letter BEC-FBS-QAU2021-347 (Dated: 03-01-2022). The healthy human participants gave their informed consent to take part in the investigation. Blood samples were taken in accordance with WHO recommendations for drawing blood (WHO, 2010). Pakistan’s National Institute of Health’s ethical standards were followed in the care and use of animals.

### 2.4 Plant collection, identification, and extraction

*A. bracteosa* was collected from the premises of QAU, Islamabad, and its taxonomy was confirmed by Dr. Mushtaq Ahmed, Department of Plant Sciences, Faculty of Biological Sciences, QAU, Pakistan. The herbarium number PHM-496 was assigned to the dried voucher specimen. The aerial parts of plant material were collected, washed, shade dried, crushed to powder, and stored in an airtight container until further use. A precisely weighed plant powder (2 kg) was steeped in 1:3 ml analytical grade methanol at room temperature (20°C–25°C) for 72 h, and the mixture was extracted by maceration and frequent sonication at 25°C (frequency 25 kHz; two cycles; 30 min) for 24 h. The marc was vacuum dried at 45°C, concentrated using a rotary evaporator (Buchi, Switzerland), and the extract was kept at −20°C (Khurshaid et al., 2020).

### 2.5 Reversed-phase high pressure liquid chromatography (RP-HPLC) based quantification of Ajugarin I in *A. bracteosa*

A solution of 20 mg/ml *A. bracteosa* and 100 μg/ml Ajugarin I was prepared by dissolving substances in HPLC-grade methanol, sonicated, centrifuged, and filtered through Sartolon polyamide membrane filters. Samples were eluted at the flow rate of 1 ml/min with injection volume, 50 μL by the means of Agilent Chem Station Rev. B. 02–01-SR1 (260) and Agilent 1,200 series binary gradient pump, accompanied by diode array detector (DAD) (Agilent technologies, Germany). Reversed-phase column chromatography was conducted using a Zorbax-C8 analytical column (4.6 × 250 mm, 5 µm particle size) from Agilent Technologies, Germany. The separation process involved a gradient of concentrations, specifically 10% methanol in water (designated as mobile phase A) and 100% methanol (designated as mobile phase B). A gradient volume of pure methanol (mobile phase B) in 0–20 min was 0%–100%, 100% in 20–23 min, and 0% for the last 23–27 min. Chromatogram was obtained at 220 nm. The concentration of standard was 100 ppm in methanol. The amount of Ajugarin I quantified per mg *A. bracteosa* was observed in µg of DW. The standard and sample were compared using retention time and comparative chromatograms.

### 2.6 RP-HPLC based quantification of polyphenols in *A. bracteosa*

Reversed phase high pressure liquid chromatography was used for polyphenolic analysis of *A. bracteosa* (Mohsin et al., 2022). A Zorbax-C8 analytical column (5 µm particle size, 4.6 cm, and 250 nm) coupled with a diode array detector (Agilent technologies, Germany) was fitted in a HPLC system (Agilent Chem station Rev. B. 02-01-SRI 260). The binary gradient solvent system comprising mobile phase A (methanol: water: acetic acid: acetonitrile in ratio of 10:85:1:5) and mobile phase B (acetonitrile: methanol: acetic acid, 40:60:1) was employed for polyphenols detection. A flow rate of 1 ml per minute was maintained and 20 µL of *A. bracteosa* (20 mg/ml) in methanol was injected. A gradient volume of mobile phase B, 0%–75% for 0–30 min, 75%–100% for 30–31 min, and 100% for the last 31–35 min, was set for elution. The samples were centrifuged and filtered before being added to the HPLC apparatus; stock solutions for all standards were freshly prepared in methanol. Mobile phases and standard solutions were first degassed and then filtered through 0.45 µm Millex-HV membrane filters.

A specific wavelength was used to measure the absorption. Polyphenols were acknowledged by comparing retention periods with reference standards as a guide to those of standards: vanillic acid, plumbagin, and thymoquinone (257 nm), catechin, coumaric acid, syringic acid, and gallic acid (279 nm), apigenin, caffeic acid, gentisic acid, and luteolin (325 nm), and quercetin, kaempferol, and myricetin (368 nm).

### 2.7 Comparative *in vitro* analysis of Ajugarin I and *A. bracteosa*

#### 2.7.1 Evaluation of antioxidant activity

Antioxidant activity for the tested samples was evaluated followed by the protocols (Zahra et al., 2017) for free radical scavenging assay (FRSA), ferric reducing antioxidant power (FRAP), and total antioxidant capacity (TAC) using ascorbic acid as positive control. Ascorbic acid equivalents per milligram dry weight of the sample was calculated for FRAP and TAC assays.

a) FRAP

Ajugarin I and *A. bracteosa* (2 mg/ml; 100 µL) were poured into Eppendorf tubes followed by the addition of 200 µL 0.2 M phosphate buffer (pH 6.6) and 250 µL of 1% potassium ferricyanide at 50°C for 20 min. After incubation, 200 µL trichloroacetic acid (10%) was added to the reaction mixture and centrifuged (3,000 rpm) for 10 min. In a 96-well plate, a volume of 150 µL of supernatant and 50 µL of FeCl_3_ was added. A microplate reader (Elx 800; Biotech United States) was used to evaluate the absorbance at 700 nm.

b) TAC

Aliquots of 100 µL of Ajugarin I and *A. bracteosa* (2 mg/ml) were mixed with 900 µL of TAC reagent (0.247 g ammonium molybdate, 1.67 g sodium monobasic phosphate, and 1.63 ml sulfuric acid added in 50 ml distilled water). Samples were then incubated for 90 min at 95°C. Absorbance of the reaction mixture was measured at 630 nm using a microplate reader.

c) FRSA

Ajugarin I and *A. bracteosa* (4 mg/ml; 10 µL) were added to a 96-well plate followed by the addition of 190 µL of DPPH. Ascorbic acid (1 mg/ml; 10 µL) was used as positive control. The plate was incubated in the dark at 37°C for an hour and absorbance was measured with a microplate reader (515 nm). FRSA was calculated using the formula:

$$\%\;Scavenging=1-\frac{Abs}{Abc}*100$$

where Ab_s_ = sample absorbance and Ab_c_ = absorbance of negative control.

#### 2.7.2 Antibacterial activity

Antibacterial assay was performed using the broth dilution method as described in the literature (Wiegand et al., 2008). It was observed against gram positive bacteria *Bacillus subtilis* (ATCC-6633) and *Staphylococcus aureus* (ATCC-6538) and gram negative strains including *Escherichia coli* (ATCC-25922), *Pseudomonas aeruginosa* (ATCC-15442), and *Klebsiella pneumonia* (ATCC-1705). All bacterial strains used in the assay were refreshed in nutrient broth 1 day before performing the assay. The turbidity of the inoculum was adjusted using a 0.5 McFarland scale. Ciprofloxacin at a concentration of 10 μg/ml was used as positive control. Stock solutions of 20 mg/ml of both Ajugarin I and *A. bracteosa* were prepared in methanol and two-fold serial dilutions of both were used in the assay. A volume of 5 µL of sample and 195 µL of inoculum was pipetted into 96-well plates. The plates were incubated for 24 h at 37°C and the absorbance was determined at 600 nm before and after incubation. Percent growth inhibitions and minimum inhibitory concentration (MIC) values were determined for both samples.

#### 2.7.3 Brine shrimp lethality assay

The standard protocol reported in the literature was followed to carry out brine shrimp lethality assay (Ul-Haq et al., 2012). Doxorubicin (40 μg/ml) was employed as positive control Ajugarin I and *A. bracteosa* were serially diluted in two-fold concentrations, i.e., 200, 100, 50, and 25 μg/ml. Percent mortality was calculated using the formula:

$$\%\;Mortality=\frac{Number\;of\;shrimps}{Total\;number\;of\;shrimps}*100$$

#### 2.7.4 Hemolytic assay

Hemolytic assay was used to analyze the interaction of Ajugarin I (200 µg) and *A. bracteosa* (200 µg) with red blood cells with slight modifications according to the previously described protocol (Waseem et al., 2023). Blood was taken in EDTA tubes and centrifuged (3,000 rpm) for 15 min after receiving volunteers’ informed consent. Supernatant was discarded out and the red blood cells pellet was rinsed 3X with PBS. A 5% suspension of the pellet in PBS was prepared. Both Ajugarin I and *A. bracteosa* were mixed with cell suspension and incubated at 37°C for 30 min. One percent Triton X was used as a positive control. Later, samples were centrifuged for 10 min (3,000 rpm), A volume of 200 µl from the supernatant was transferred to 96-well plates, and the absorbance was measured at 541 nm. The percent hemolytic activity was calculated using the formula:

$$\%\;Hemolysis=\frac{\left(Absorbance\;of\;sample-Absorbance\;of\;negative\right)}{\left(Absorbance\;of\;positive-Absorbance\;of\;negative\right)}$$

### 2.8 *In vivo* wound healing activity

#### 2.8.1 Animals and groups

Balb/c mice (25–30 g) aged 6–7 weeks were used in the full thickness excision wound model. Mice were habituated to the laboratory environment before experiment execution. After ethical approval (BEC-FBS-QAU2021-347 dated: 03-01-2022), five groups (n = 6) of mice were formed. Group I untreated, Group II vehicle control (10% ethanol), and Group III served as positive control with applications of Povidone-iodine. Groups IV and V were treated with Ajugarin I and *A. bracteosa,* respectively. The sample’s stock solution 1% (w/v) was prepared with10% (v/v) ethanol in distilled water.

#### 2.8.2 Dermal irritability test

Mice (n = 6) were initially subjected to dermal irritability test to assess the topical toxicity of Ajugarin I and *A. bracteosa*. A patch of skin from the backside of each mouse was shaved and Ajugarin I (200 µg) and *A. bracteosa* (200 µg) solutions were applied to the skin. After 24 h of application, the presence of erythema and edema on the target skin area was observed (Gebrehiwot et al., 2015).

#### 2.8.3 Excision wound model

The excision wound model was used to evaluate the wound healing ability of Ajugarin I and *A. bracteosa* (Das, 2013). Mice (n = 6) were given 60 µL of analgesic tramadol (10 mg/kg; Searle (Pvt.) Ltd., Pakistan) at least 1 h before the wound incision. A hair removal cream was then used to shave the mice’s backs between their shoulder blades. The area was locally anesthetized with lidocaine gel and mice were anesthetized by exposure to chloroform for 5 s. Skin was swabbed with alcohol and a small biopsy punch was used to make an excision wound on the dorsal surface of the skin, 5 mm in diameter. After excision, the mice were stabilized from the effect of anesthesia and wound size was measured as a day 0 reading. Ajugarin I and *A. bracteosa* (200 µg/20 µL each) were applied on the wound site. Likewise, Povidone-iodine and 10% ethanol were applied as controls to the respective groups. The following parameters were observed for the duration of study.

#### 2.8.4 Wound contraction measurement

Wound size was measured by tracing the wound on a transparent sheet of paper on alternate days (i.e., day 0, 2, 4, 6, 8, 10, 12, and 14) until the wound was completely recovered for at least one group. The percentage of wound contraction was calculated using the formula:

$$\%\;Wound\;contraction=A0-\frac{At}{A0}*100$$

where A0 is the area of the original wound and At is the area of the wound at the particular day of measurement.

#### 2.8.5 Chromatic study

A chromatic code was given to wounds as described previously (Trabelsi et al., 2017). These codes are bright red, dark, red, and pink for blood on the wound, the coagulation of blood, granulation tissue formation, and epithelialization process, respectively.

#### 2.8.6 Epithelialization time

The time required for dead tissue to shed without any visible raw wound was used to determine how long epithelization lasted. After the injury, the epithelialization process was tracked up to 20 days (Tekleyes et al., 2021).

#### 2.8.7 Histopathological analysis

Skin from the wound area was excised and then fixed within 10% formalin. H&E stained slides of skin biopsy were observed using a light microscope to evaluate morphological changes during the wound healing process among the different animal groups (Ahmad et al., 2023).

### 2.9 Statistical analysis

All findings were depicted as mean ± standard deviation of respective parameters. The statistical analysis and graphical representation were done using 2D v5.01 Table Curve and Origin Pro 2018, respectively. The statistical significance (*p* < 0.05) among the groups was assessed using ANOVA with Bonferroni’s posttest.

## 3 Results

### 3.1 Qualitative and quantitative analysis of Ajugarin I in *A. bracteosa*

**FIGURE 1:**
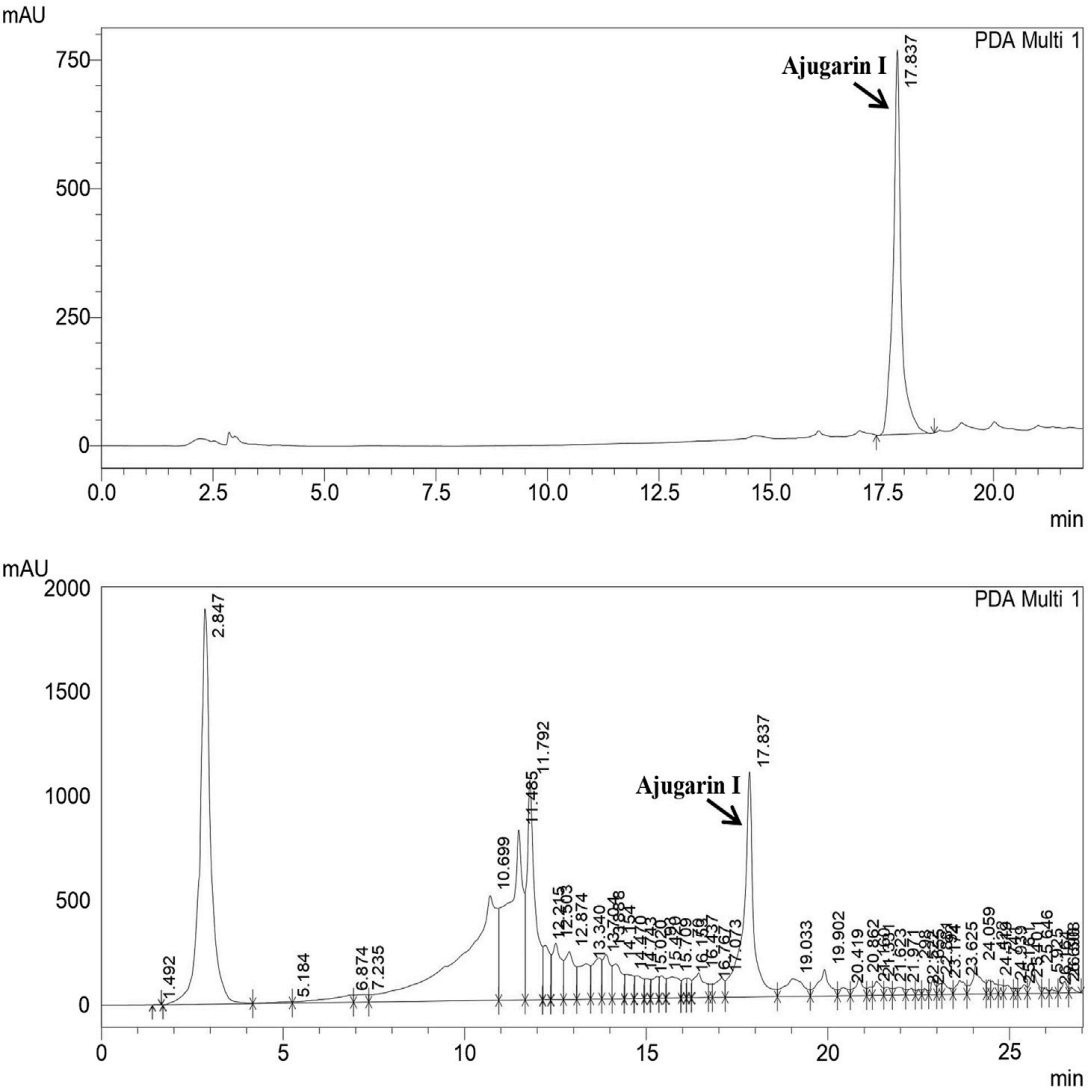
Quantification of Ajugarin I in *A. bracteosa* using reversed phase high-performance liquid chromatography (RP-HPLC). Chromatogram of *A. bracteosa* (bottom) was compared with the standard pure Ajugarin I chromatogram (top). Ajugarin I was quantified by comparing the area under curve (AUC) in both chromatograms.

RP-HPLC confirmed and quantified the amount of Ajugarin I in *A. bracteosa* samples (Figure 1). The chromatogram of standard Ajugarin I showed its peak (83,717) at the retention time of 17.83 min. A similar peak was observed in *A. bracteosa* samples, indicating the presence of Ajugarin I with an amount of 2.2 ± 0.02 μg/mg DW (Figure 1).

### 3.2 Quantification of polyphenols in *A. bracteosa*

**FIGURE 2:**
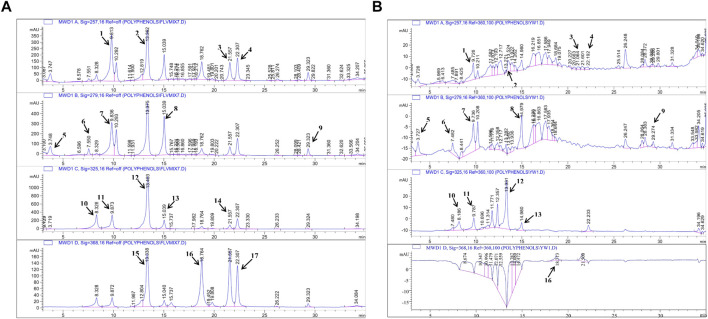
Quantification of polyphenols in *A. bracteosa.* Polyphenolic compounds were quantified in *A. bracteosa* using RP-HPLC. Chromatograms of 17 polyphenol standards **(A)** were compared with the chromatogram of *A. bracteosa* samples **(B)**. Each numbered peak represents a phenolic compound, i.e., vanillic acid (1), rutin (2), plumbagin (3), thymoquinone (4), gallic acid (5), catechin (6), syringic acid (7), coumaric acid (8), emodin (9), gentisic acid (10), caffeic acid (11), ferulic acid (12), cinnamic acid (13), apigenin (14), myristicin (15), quercetin (16), and kaempferol (17).

In the next step, the presence of polyphenols in *A. bracteosa* was examined via the RP- HPLC method to ensure the presence of phytoconstituents other than Ajugarin I. Herein, chromatographs of *A. bracteosa* were compared with standards for the quantitative measurement of polyphenols (Figure 2A). Among 17 tested polyphenols, *A. bracteosa* was found to contain vanillic acid, thymoquinone, rutin, plumbagin, quercetin, gallic acid, catechin, coumaric acid, syringic acid, caffeic acid, emodin, gentisic acid, cinnamic acid, and ferulic acid. Three compounds including apigenin, myristicin, and kaempferol were not detected in *A. bracteosa*. Catechin (0.38 ± 0.014 μg/mg extract of *A. bracteosa*) was found in highest concentration compared to other polyphenols (Figure 2B; Table 1).

**TABLE 1:** Quantification of polyphenols in *A. bracteosa.*

Sr no.	Phenols	Signal wavelength (nm)	Retention time (min)	Concentration (µg/mg extract of *A. bracteosa*)±SD
1	Vanillic acid	257	9.8	0.113 ± 0.02
2	Rutin	257	13.2	0.004 ± 0.019
3	Plumbagin	257	21.5	0.046 ± 0.003
4	Thymoquinone	257	22.3	0.047 ± 0.001
5	Gallic acid	279	3.7	0.180 ± 0.015
6	Catechin	279	7.5	0.381 ± 0.014
7	Syringic acid	279	9.8	0.135 ± 0.008
8	Coumaric acid	279	15.03	0.101 ± 0.030
9	Emodin	279	29.3	0.106 ± 0.02
10	Gentisic acid	325	8.3	0.062 ± 0.002
11	Caffeic acid	325	9.8	0.064 ± 0.002
12	Ferulic acid	325	13.4	0.075 ± 0.002
13	Cinnamic acid	325	15	0.071 ± 0.001
14	Apigenin	325	21.5	Not detected
15	Myricitin	368	13.3	Not detected
16	Quercitin	368	18.7	0.042 ± 0.003
17	Kaempferol	368	22.3	Not detected

### 3.3 *In vitro* assessment of biological profile

#### 3.3.1 Evaluation of antioxidant activity

**FIGURE 3:**
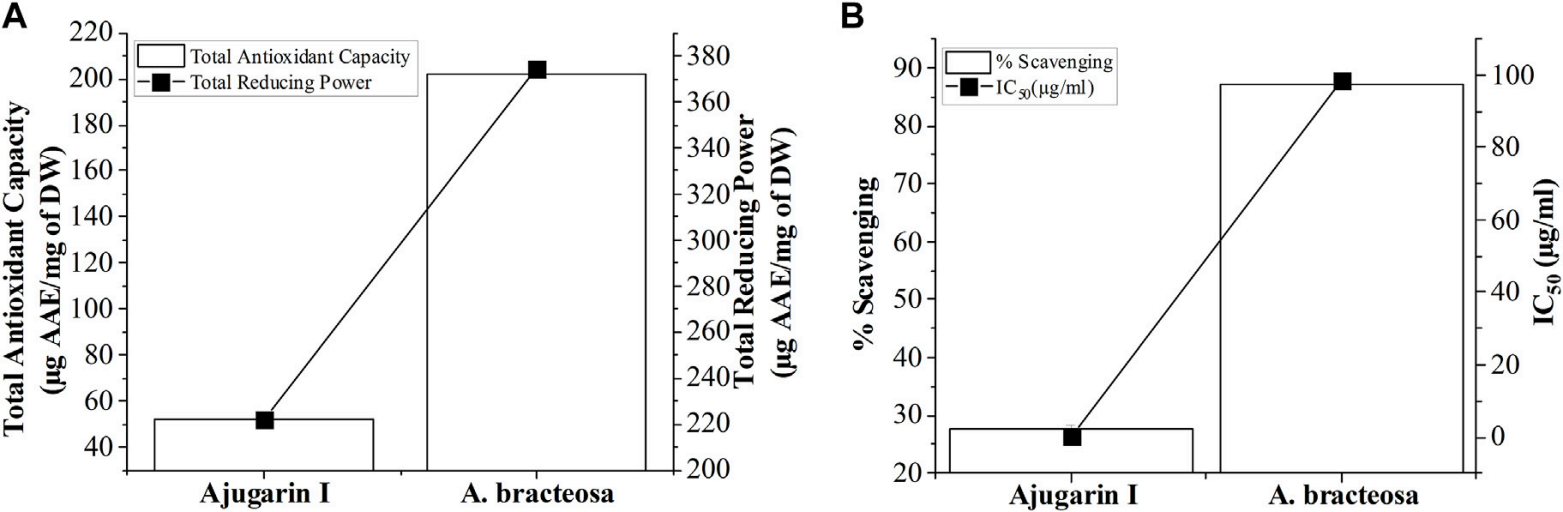
Graphical representation of antioxidant activity. The antioxidant potential of *A. bracteosa* and Ajugarin I were evaluated using three assays. **(A)** Total antioxidant capacity and total reducing power assays showed a higher antioxidant capacity of *A. bracteosa* than Ajugarin I. Data are presented as micrograms of ascorbic acid equivalent (AAE) per milligrams of dry weight (DW) of samples. **(B)** Free radical scavenging assay also depicted a better antioxidant profile of *A. bracteosa* than Ajugarin I. Assays were performed using DPPH. IC_50_: 50% inhibitory concentration.

We subsequently established the antioxidant profile of *A. bracteosa* and Ajugarin I. Results showed that *A. bracteosa* exhibited higher antioxidant activity than Ajugarin I as detected by FRAP, TAC, and FRSA (Figure 3; Table 2). The FRAP colorimetric assay showed a 1.86-fold greater antioxidant activity of *A. bracteosa* in contrast to Ajugarin I with values of 374.48 ± 0.5 and 221.87 ± 0.2 µg AAE/mg DW, respectively. Likewise, the TAC assays demonstrated a 3.89-fold greater antioxidant capacity of *A. bracteosa* than Ajugarin I. The activity was quantified as 51.81 ± 0.4 and 201.95 ± 0.2 µg AAE/mg DW for Ajugarin I and *A. bracteosa*, respectively (Figure 3A; Table 2). Antioxidant activity was further endorsed by the FRSA that utilized the change in the color of DPPH when scavenged by antioxidant constituents. The FRSA value for *A. bracteosa* was 3.15-fold higher than Ajugarin I (Table 2) at 200 μg/ml. *A. bracteosa* showed strong antioxidant potential with an IC_50_ value of 78.53 ± 0.6 μg/ml (Figure 3B).

**TABLE 2:** Antioxidant potential and brine shrimp cytotoxicity of Ajugarin I and *A. bracteosa*.

Samples	Antioxidant potential			Brine shrimp cytotoxicity				
	TAC (µg AAE/mg of DW)	FRAP (µg AAE/mg of DW)	FRSA (%)	Mortality (%)				
				200 (µg/ml)	100 (µg/ml)	50 (µg/ml)	25 (µg/ml)	LC_50_ (µg/ml)
Ajugarin I	51.8 ± 0.40	221.8 ± 0.50	27.65 ± 0.80	56.6 ± 5.77	36.6 ± 5.77	26.6 ± 5.77	6.66 ± 5.77	70.71
*A. bracteosa*	201.9 ± 0.20	374.4 ± 0.20	87 ± 0.30	36.6 ± 5.77	16.6 ± 5.77	6.67 ± 5.77	--	>200
Doxorubicin	NA	NA	NA	96.6 ± 5.77	90.0 ± 0.00	60.0 ± 0.00	40.0 ± 0.00	5.63 ± 0.25
DMSO	NA	NA	NA	--	--	--	--	--

***Note:*** Data are presented as mean ± SD, of respective parameters; AAE, ascorbic acid equivalent; Negative control = DMSO; -- = No activity; NA, Not applicable.

#### 3.3.2 Assessment of antibacterial activity

We assessed the antibacterial action of Ajugarin I and *A. bracteosa* against five non-resistant strains by measuring the percent growth inhibition of bacteria and MIC values. It was observed that *A. bracteosa* inhibited the growth of *S. aureus* (91.11 ± 0.89%; MIC = 25 μg/ml), *B. subtilis* (92.59 ± 0.74%; MIC = 200 μg/ml), and *E.coli* (89.97 ± 2.10%; MIC = 200 μg/ml) while Ajugarin I showed activity against *S. aureus* (89.81 ± 1.04%; MIC = 50 μg/ml) and *B. subtilis* (90.37 ± 0.43%; MIC = 200 μg/ml) only (Table 3). These results were insignificantly (*p* > 0.05) different from the standard ciprofloxacin that showed 92.96 ± 0.69, 92.90 ± 1.03, and 93.70 ± 0.53% bacterial growth inhibition against *S. aureus*, *E.coli*, and *B. subtilis*, respectively.

**TABLE 3:** Antibacterial and hemolytic activities Ajugarin I and *A. bracteosa*.

Samples		Minimum inhibitory concentration (μg/ml)				Hemolysis (%)
	*S. aureus*	*B. subtilis*	*P. aeruginosa*	*K. pneumonia*	*E. coli*	
Ajugarin I	50	200	--	--	--	1.45 ± 0.25
*A. bracteosa*	25	200	--	--	200	1.81 ± 0.09
Ciprofloxacin	1.25	1.25	1.25	1.25	1.25	NA
DMSO	--	--	--	--	--	NA
Triton X	NA	NA	NA	NA	NA	100 ± 0.001
Normal saline	NA	NA	NA	NA	NA	0

***Note:*** - = Not active; sample concentration = 200 μg/ml; NA , Not applicable; positive and negative controls in antibacterial assays were ciprofloxacin (10 μg/ml) and DMSO, respectively, while in hemolytic assays, triton X and normal saline were used as positive and negative control, respectively. Data are presented as mean ± SD, of percent hemolysis.

#### 3.3.3 Cytotoxicity assessment by brine shrimp lethality assay

Our results showed that Ajugarin I was more toxic to brine shrimp larvae with 56.6 ± 5.77% mortality at 200 μg/ml. The LC_50_ value for Ajugarin I was estimated as 70.71 μg/ml. On the contrary, *A. bracteosa* was 1.54-fold less toxic than Ajugarin I with mortality and an LC_50_ value of 36.6 ± 5.77% and >200 μg/ml, respectively (Table 2).

#### 3.3.4 Determination of *in vitro* hemolytic activity

It was found that both Ajugarin I and *A. bracteosa* exhibited less than 2% hemolysis, which deemed both samples non-hemolytic and thus safe to use (Table 3). The results were significant as *p* < 0.05, in comparison to positive control.

### 3.4 *In vivo* assessment of wound healing profile

#### 3.4.1 Estimation of safety profile by dermal irritability test

The safety profile of *A. bracteosa* and Ajugarin I was further assessed using an *in vivo* acute dermal irritability test on mice. All animal (n = 6) groups exhibited no symptoms of inflammation, edema, redness, or irritation after topical application of Ajugarin I (200 µg) and *A. bracteosa* (200 µg) for a period of 24 h. Ajugarin I and *A. bracteosa* were rendered free of any irritants or harmful compounds at the test dose because there was no evidence of any illness, physical, or weight fluctuations.

#### 3.4.2 Wound contraction measurement

**FIGURE 4:**
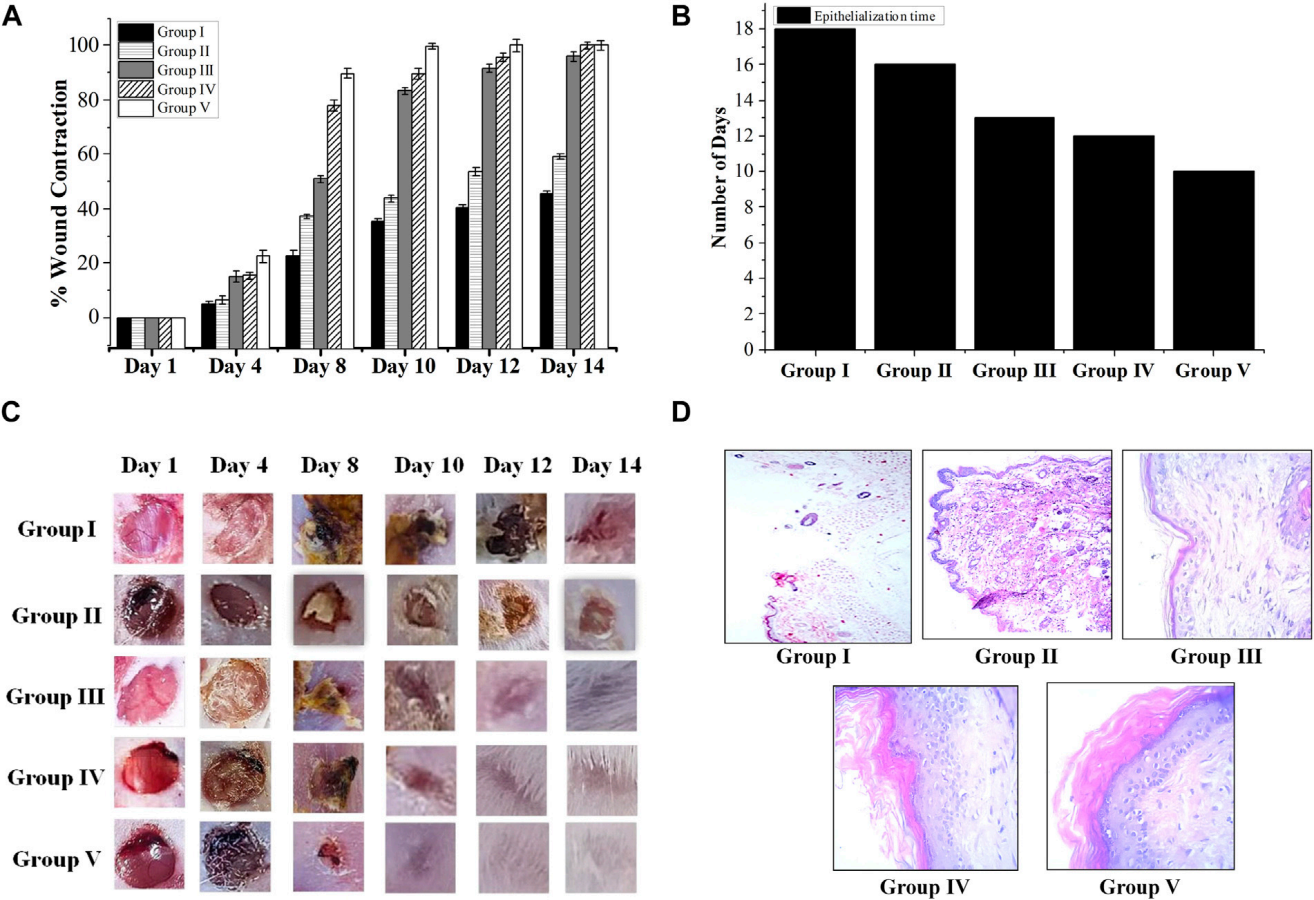
Wound healing capacity of *A. bracteosa* and Ajugarin I. Mice (n = 6) were inflicted with superficial wounds and divided into five groups. Group I = untreated group, Group II = vehicle (10% ethanol) control group, Group III = positive control group (Povidone-iodine), Group IV = treated with Ajugarin I, and Group V = treated with *A. bracteosa*. Data are mean ± SD of respective parameters. **(A)** Percent wound contraction in response to different treatments. The wound area was measured for 14 days until the wound in one of the groups was completely healed. **(B)** Epithelization time in days taken by the wounds in response to different treatments. **(C)** Chromatic analysis where wounds were visually observed for macroscopic changes. **(D)** H&E stained 5 mm thick section of skin biopsies at the site of wound that are photomicrographs at 40X depicting the microscopic changes during wound healing in response to different treatments.

Ajugarin I (200 µg) and *A. bracteosa* (200 µg) were assessed in an excision wound model in mice (n = 6) to compare their wound healing activity. Results showed noteworthy (*p* < 0.05) wound healing activity of both Ajugarin I and *A. bracteosa* as compared to Group II (10% ethanol; vehicle control) and Group III (Povidone-iodine; positive control). Apparently, there was no discernible difference in the activity of Ajugarin I and *A. bracteosa* on day 14 of the treatment, yet it was 1.68-fold higher than the vehicle control. On the contrary, *A. bracteosa* (100 ± 2.34%) healed the wound completely and 1.04-fold more efficiently than Ajugarin I (95.6 ± 1.52%) until day 12 of the treatment (Table 4; Figure 4A).

**TABLE 4:** Effect of different treatments on wound contraction and epithelialization time.

	Day 1	Day 4	Day 8	Day 10	Day 12	Day 14	
Group I	0	4.8 ± 1.30	22.8 ± 1.70	35.16 ± 0.98	40.44 ± 1.02	45.5.±0.93	17.6 ± 0.47
Group II	0	6.37 ± 1.64	37.16 ± 0.87	43.8 ± 1.24	53.44 ± 1.56	59.2 ± 0.91	15.3 ± 0.47
Group III	0	15.04 ± 2.08	50.88 ± 1.12	83.28 ± 1.45	91.64 ± 1.67	96 ± 1.90	13.6 ± 0.47
Group IV	0	15.36 ± 1.07	78 ± 1.90	89.59 ± 1.94	95.6 ± 1.52	100 ± 1.45	11.6 ± 0.47
Group V	0	22.56 ± 2.12	89.76 ± 1.67	99.9 ± 1.08	100 ± 2.34	100 ± 1.78	9.33 ± 0.47

***Note:*** Data are presented as mean ± SD, of respective parameters. Group I = untreated group, Group II, vehicle control group (10% ethanol), Group III, treated with Povidone-iodine, Group IV, treated with Ajugarin I, Group V = treated with *A. bracteosa*.

Likewise, *A. bracteosa* (99.9 ± 1.08%) was 1.11-fold more active than Ajugarin I (89.59 ± 1.94%) on day 10. Concisely, on days 10 and 12, *A. bracteosa* exhibited a faster wound healing profile than Ajugarin I with values of 2.28-, 1.87-folds and 2.04-, 1.78-folds, respectively higher in relation to vehicle control (10% ethanol) (Table 4; Figure 4A).

#### 3.4.3 Chromatic study and epithelization time

The epithelialization time is the time needed by the wound to completely heal. Epithelization period was monitored for 20 days after treatment. It was noticed that the pace of wound closure accelerated with each day of treatment in the group treated with *A. bracteosa*, indicating a shorter time of epithelization. *A. bracteosa* (9.33 ± 0.47 days) demonstrated the fastest period of epithelization followed by Ajugarin I (11.6 ± 0.47 days), which were 1.63- and 1.31-fold higher than 10% ethanol treated (15.3 ± 0.47 days) group, respectively. Moreover, epithelization time was also 1.45-fold more rapid in the *A. bracteosa* treatment group than Povidone-iodine treated group (13.6 ± 0.47 days) (Figure 4B; Table 4). Additionally, we visually examined the wound for macroscopic changes. It was observed that the wound was bright red in color due to enhanced blood flow on the first day in all groups of mice. Gradually, a brown scab was quickly formed in Ajugarin I (Group IV) and *A. bracteosa* (Group V) treated mice as compared to 10% ethanol (Group II; vehicle control) or Povidone-iodine (Group III; positive control) treated mice. The macroscopic examination indicated that Group V showed better reconstruction of the wound. Cellular debris detached from the wound with the appearance of a healthy light pink color on days 8 and 10 for *A. bracteosa* and Ajugarin I, respectively (Figure 4C). Wounds in the mice of Group V (*A. bracteosa* treated) were completely closed on day 12 with the formation of granulation tissue. On the contrary, Povidone-iodine (Group II; positive control) treated wounds turned light pink on day 12 and were not completely healed until that day (Figure 4C). Thus, the results indicated the effective wound healing capacity of Ajugarin I and *A. bracteosa* with comparatively more efficient activity in *A. bracteosa*.

#### 3.4.4 Histopathological analysis

Histopathological analysis of skin removed from the wound was done on the day 14 for supplementary validation of the effectiveness of samples in relation to revascularization, epithelization, and fibrosis within the dermis and a noticeable layer of the epidermis. Microscopic examination of mice treated with *A. bracteosa* and Ajugarin I revealed increase production of fibroblasts (Figure 4D). This indicates that these samples might have enhanced the contractile functions of myofibroblasts, prompting earlier wound contraction (Dunn et al., 2013). *A. bracteosa* and, Ajugarin I cause remodeling of dermal and epidermal layers faster than positive and vehicle control groups.

Furthermore, *A. bracteosa* treated wounds demonstrated better epithelialization, distinct thick dermal/epidermal layers, and notable keratinization as compared to Ajugarin I, positive, and vehicle controls. The keratin layer in positive controls (Group III) was very thin and loosely attached. Likewise, the keratin layer in Ajugarin I (Group IV) group was also partially loosely attached as compared to *A. bracteosa* group (Group V) (Figure 4D). On the other hand, the untreated and vehicle control groups showed no definite epidermal and dermal layers with either no or very thin keratin layers.

*A. bracteosa* and Ajugarin I treated wounds exhibited spindle-shaped fibroblasts that were well proliferated and disseminated in granulation tissue. These fibroblasts were comparatively fewer in number in the Ajugarin I group as compared to the *A. bracteosa* group. *A. bracteosa* and Ajugarin I accelerated the healing of the wound by stimulating angiogenesis, keratin synthesis, significant fibroblast activity, and collagen synthesis. The lumina formations, which are surrounded by flat endothelial cells, were used to identify new blood arteries. A significant number of extra vessels were found to be distributed in the granulation tissue of *A. bracteosa* treated wounds (Figure 4D). The tissue samples from the vehicle control, on the other hand, showed fewer new vessels.

## 4 Discussion

Wounds are everyday occurrences that can be treated with allopathic or traditional remedies. Most often traditional remedies preferably with anti-inflammatory and antimicrobial properties are used to decrease inflammation and prevent the infection of wounds (Dunn et al., 2013). Recently, it was discovered that *Ajuga bracteosa* possesses substantial wound healing properties. It is yet unknown, nevertheless, whether the plant’s effectiveness is linked to Ajugarin I alone or also involves other phytoconstituents. Therefore, the current investigation was conducted to report the comparative wound healing profiles of Ajugarin I and *A. bracteosa* powder.

Qualitative and quantitative analysis of Ajugarin I in *A. bracteosa* revealed our results are in consent with Singh et al., who characterized Ajugarin I in the hexane extract of *A. bracteosa* (Singh et al., 2006). Additionally, Khan et al. have reported that Ajugarin I can reduce neuropathic pain associated with diabetic neuropathy by augmenting antioxidant levels and reducing inflammatory cytokines (Khan et al., 2022). Thus, Ajugarin I is a bioactive compound detected in *A. bracteosa* in our study.

Polyphenols have well established pharmacological profiles (Xie et al., 2019). For example, emodin, myricetin, coumaric acid, and rutin exhibit antioxidant, anti-inflammatory, and antibacterial activities (Dai and Mumper, 2010). Ferulic acid showed good anti-inflammatory, neuroprotective, and antidiabetic activities (Gohil et al., 2012). Prolonged oxidative stress leads to atherosclerosis, degenerative disease, and cancer (Muhammad et al., 2021). Gallic acid has been reported as a shielding gadget to the body from oxidative damage and ultimately can cure a disease (Gao et al., 2019). Moreover, catechin was found to be effective against inflammation, cancer, and arthritis and also to possess bactericidal properties (Baranwal et al., 2022). Epidemiological findings also showed a strong relationship between the consumption of a phenolic acid-enriched diet and the prevention of diseases (Ashokkumar et al., 2013).

Oxidative stress is the root of various ailments such as neurodegenerative and cardiovascular disorders, inflammatory diseases, and cancer. Plants synthesize a variety of antioxidants, due to which two-thirds of plant species are considered to be therapeutically important owing to their significant antioxidant properties (Kasote et al., 2015). Reactive oxygen species (ROS) generated by polymorphonuclear cells in the vicinity of wounds can damage the endothelial membrane and impede the process of healing (Bryan et al., 2012). Natural antioxidants can detoxify and counteract free radicals, protecting cells from oxidative stress-induced injuries (Rauf et al., 2023). Antioxidants facilitate wound healing by mitigating oxidative stress-induced cellular damage, promoting tissue repair, and enhancing key processes such as collagen synthesis and angiogenesis. Consequently, antioxidant compounds benefit the wound healing process. Similarly, *Ajuga reptans* (Toiu et al., 2017)*, Ajuga genevensis*, and *Ajuga salicifolia* (Göger et al., 2021) have been reported to possess substantial antioxidant activities. Certain bioactive constituents within plants possess unbound hydroxyl (−OH) groups, which exhibit antioxidant activity (Zahra et al., 2017). Ajugarin I is a neo-clerodane diterpenoid that lacks a free hydroxyl group and thus demonstrates a low *in vitro* antioxidant profile as compared to *A. bracteosa*. Remedies with good antibacterial profiles will be beneficial in preventing infection of wounds. The literature shows that phytochemicals in medicinal plants are effective in impeding the growth of both gram positive and gram negative bacteria (Satish et al., 2008). Another plant from *Ajuga* species known as *Ajuga reptans* also exhibited antibacterial activity against *S. aureus* and *E. coli* (Toiu et al., 2017). Additionally, *Ajuga lupulina* was found to possess neoclerodane diterpenoids, which were related to its antibacterial activity (Coll and Tandrón, 2008). Since Ajugarin I is also a diterpenoid, Coll and Tandron’s claim therefore supports our results. Additionally, phenols, tannins, and flavonoids exhibit significant antibacterial effect against gram positive bacteria (Madureira et al., 2012). These could also contribute to the antibacterial profile of *A. bracteosa* as we have quantified significant polyphenols in *A. bracteosa*. Thus *A. bracteosa* exhibited higher antibacterial capacity than Ajugarin I alone, suggesting its potential effectiveness in preventing bacterial colonization at wound sites. The *in vitro* brine shrimp lethality assay is an expedient means for determining the cytotoxicity of natural products. It is an easy and inexpensive analysis which requires minimal amounts of test material. Furthermore, another feature of hindered wound restoration is helminth infections. Parasites tend to infiltrate the skin lesions (Bland et al., 1984), infecting the injury, causing pain, inflammation, and delayed healing (Bland et al., 1984). Hence, anthelmintics may be useful in preventing helminth infections and permitting unhindered wound healing by suppressing the growth of parasite larvae. Consequently, brine shrimp assay depicts both cytotoxicity against normal cells and effectiveness against helminth infection. Substances that are highly cytotoxic could have adverse effects on the cells involved in the wound healing process. They might impede the normal cellular functions, including cell migration, proliferation, and tissue remodeling, which are crucial for effective wound repair. Since Ajugarin I is pure compound, its affinity with the shrimp larvae could therefore be “greater” as compared to the *A. bracteosa* that is a mixture of complex phytochemicals. Zahra et al., have previously reported an LC_50_ of >100 μg/ml for *A. bracteosa* against brine shrimp larvae (Zahra et al., 2017). Similarly *A. parviflora* showed an LD_50_ of 321.4 μg/ml, which indicates that the plant has low cytotoxic potential (Rahman et al., 2013). Additionally, Khanavi et al. demonstrated that the hexane fraction of *Ajuga chamaecistus* subspecies *tomentella* showed larvicidal action (Khanavi et al., 2017).

Next, the cytotoxic potential of *A. bracteosa* and Ajugarin I was analyzed via hemolytic assay conducted on fresh human blood samples. The hemolytic assay uses freshly isolated red blood cells to quickly confirm the probability of the toxicity of a particular substance. The hemolytic assay is preferred as red blood cells are easy to isolate and their membrane structure resembles the membranes of other cells in body (Zohra and Fawzia, 2014). In accordance with ASTM F756-00 criteria for the evaluation of hemolytic potential of materials, samples with hemolysis percentages of >5%, <5%, and <2% are depicted as hemolytic, slightly hemolytic, and non-hemolytic, respectively (Elahi et al., 2014).

Traditionally, *A. bracteosa* was used in the treatment of swollen wounds (Ali, 2019) and was reported to possess anti-inflammatory and analgesic activities (Pal and Pawar, 2011). A recent study showed significant wound healing activity of *A. bracteosa* nanoparticles (Andleeb et al., 2022). However, there is no clear information whether this activity was particularly due to Ajugarin I or was associated with other phytoconstituents. Furthermore, the wound healing capacity of Ajugarin I has not been investigated. Hence, in the existing research, we evaluated and report the significant wound healing activity of both *A. bracteosa* and Ajugarin I. A faster rate of wound retraction results in an earlier period of epithelization and collagen deposition (Prasad et al., 2010). Furthermore, formation of a fibrin clot at the site of wound (Prasad et al., 2010) assists to safeguard the injured area from infections and prevents loss of blood. A scab forms as the clot hardens and parches, shielding the affected area (Bucur et al., 2019). The literature shows that fibroblasts bordering the wound start proliferating as soon as the injury is induced and, generally by day 4 after the injury, these fibroblasts move to the provisional matrix of the clot in the wound. This facilitates the process of fibroplasia, i.e., the formation of collagen-rich matrix, which comprises of collagens, proteoglycans, and elastin (Li et al., 2007). Increased angiogenesis and collagen synthesis build a hemostatic plug, improve blood flow to the injured area, and give vital nutrients for the epithelization process to the wound area. Phytochemicals like unsaturated sterols, flavonoids, phenolic acids, triterpenoids, and saponins are present in *A. bracteosa*, which may assist the wound healing capacity of *A. bracteosa* along with Ajugarin I (Zahra et al., 2017). Flavonoids inhibit formation of inflammatory mediators through cyclooxygenase and lipoxygenase pathways (Nunomura et al., 2009). We have established the antioxidant and antibacterial properties of *A. bracteosa* in the current investigation. There is potential correlation between these activities and the greater wound healing capacity of *A. bracteosa* in comparison to Ajugarin I alone. Furthermore, Ajugarin I seem to greatly aid the wound healing profile of *A. bracteosa*.

## 5 Conclusion

The present study provides insight into the *in vitro* antibacterial, antioxidant, cytotoxic, and hemolytic profile of *A. bracteosa* and Ajugarin I as well as their *in vivo* wound healing properties. Overall, compared to Ajugarin I, *A. bracteosa* showed better antioxidant, antibacterial, and wound healing capacities, which can be attributed to the existence of a variety of phytoconstituents in *A. bracteosa*. Both samples depicted non-toxic profiles*in vitro* hemolytic and *in vivo* dermal toxicity assays. Additionally, *A. bracteosa* exhibited faster wound contraction and shorter epithelization time with greater fibroblast propagation, collagen production, and revascularization in the wounds as compared to Ajugarin I. In conclusion, our findings support the ethnomedicinal use of *A. bracteosa* for wound healing and present Ajugarin I and *A. bracteosa* as possible potential substances for the exploration of wound healing drugs. Further investigations are suggested to isolate phytoconstituents from *A. bracteosa* and establish the molecular mechanism for the wound healing activity of Ajugarin I and *A. bracteosa*.

## Data Availability

The raw data supporting the conclusion of this article will be made available by the authors, without undue reservation.

## Abbreviations

AAE, ascorbic acid equivalent; A. bracteosa, Ajuga bracteosa; DMSO, dimethyl sulfoxide; DPPH, 2, 2-Diphenyl-1-picrylhydrazyl; FRAP, ferric reducing antioxidant power; FRSA, free radical scavenging activity; IC50, 50% inhibitory concentration; LC50, lethal concentration causing 50% mortality; MIC, minimum inhibitory concentration; TAC, total antioxidant capacity.
